# Supplementary material for: Deciphering Abnormal Platelet Subpopulations in COVID-19, Sepsis and Systemic Lupus Erythematosus through Machine Learning and Single-Cell Transcriptomics
Source: Int J Mol Sci. 2024 May 29;25(11):5941. doi: 10.3390/ijms25115941 (PMC11173046; doi:10.3390/ijms25115941)
Supplement: Supplementary file 1 [file ijms-25-05941-s001.zip › ijms-2991101-supplementary.pdf]

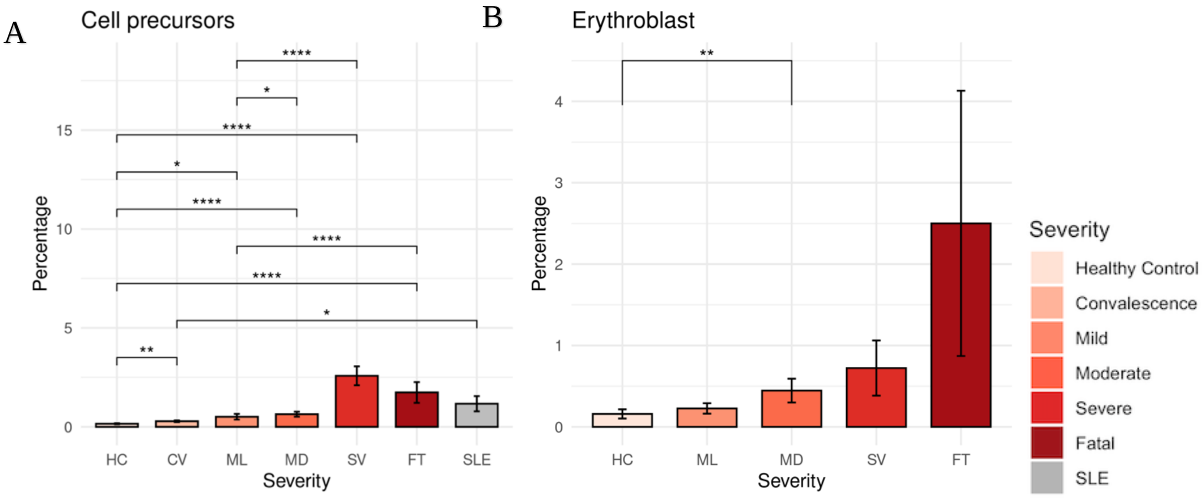

Supplementary Figure 1. **PBMC profiling from healthy controls, sepsis, similar symptom hospitalized, COVID-19 and SLE patients.** (A-C) Bar plots depicting the percentage of different cell types under different disease severities. A) Cell precursors, B) Erythroblast

The differences in percentages associated with adjusted *P*-values below 0.05, 0.01, 0.001, and 0.0001 are indicated as *, **, ***, and ****, respectively and not significant are not shown. The significance analysis was performed using Wilcoxon tests. Standard error bars were also added.

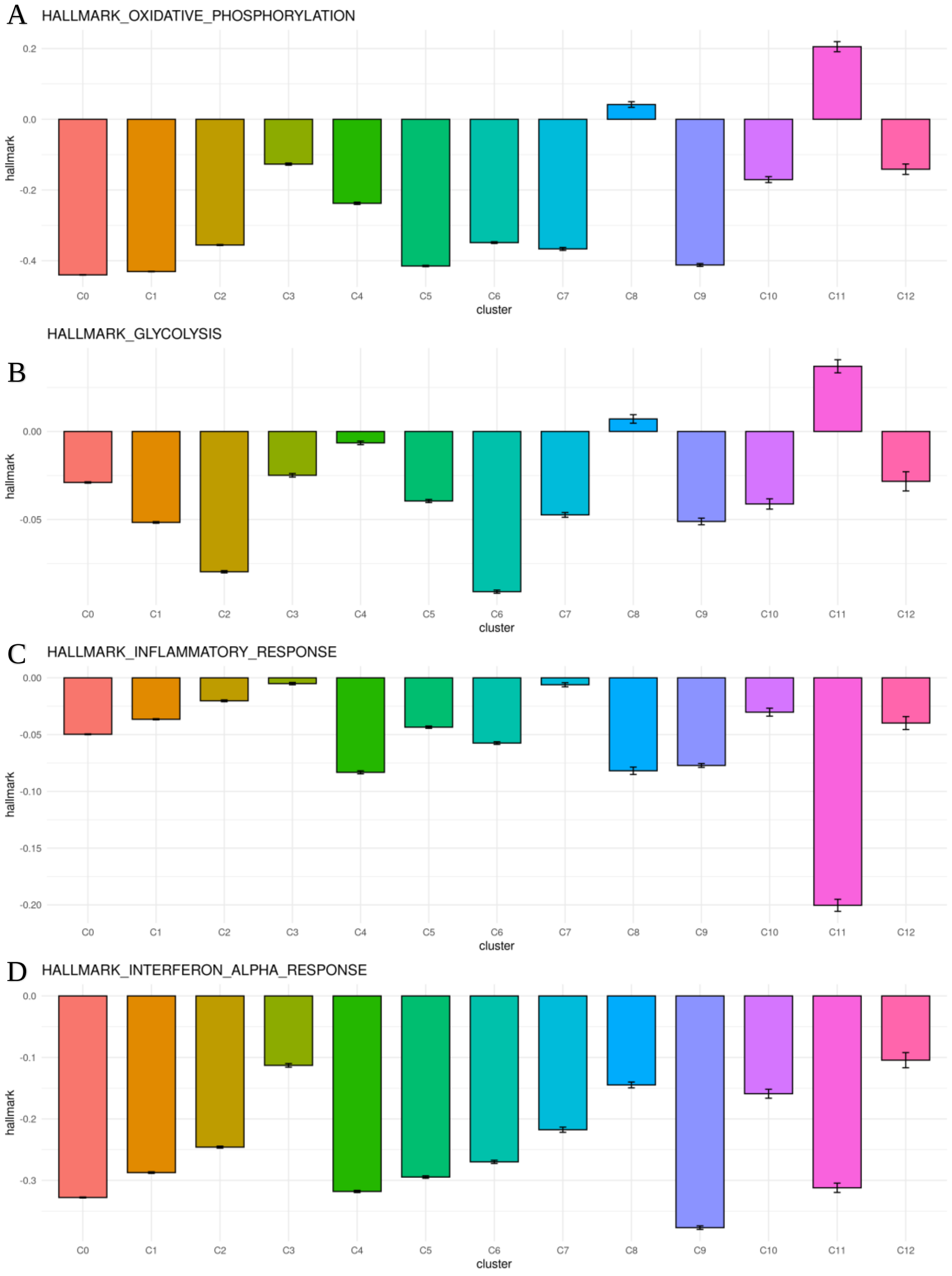

A
HALLMARK_OXIDATIVE_PHOSPHORYLATION
B
HALLMARK_GLYCOLYSIS
C
HALLMARK_INFLAMMATORY_RESPONSE
D
HALLMARK_INTERFERON_ALPHA_RESPONSE
hallmark
cluster
C0
C1
C2
C3
C4
C5
C6
C7
C8
C9
C10
C11
C12
0.2
0.0
-0.2
-0.4
0.00
-0.05
-0.10
-0.15
-0.20
-0.1
-0.3

E
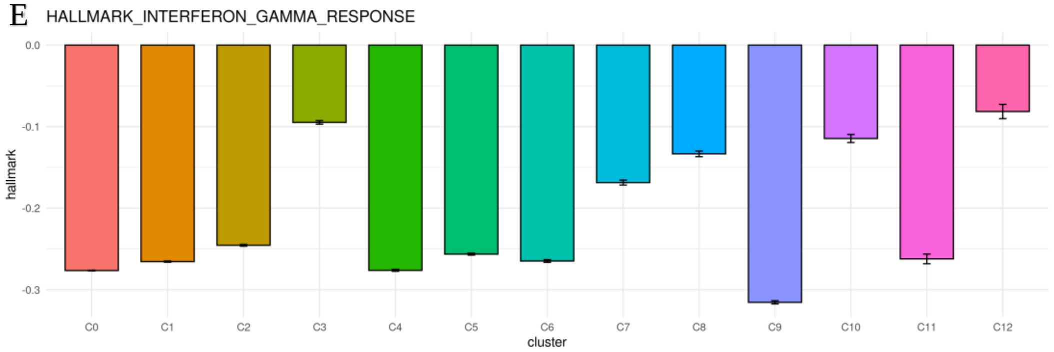
HALLMARK_INTERFERON_GAMMA_RESPONSE
hallmark
0.0
-0.1
-0.2
-0.3
C0 C1 C2 C3 C4 C5 C6 C7 C8 C9 C10 C11 C12
cluster

F
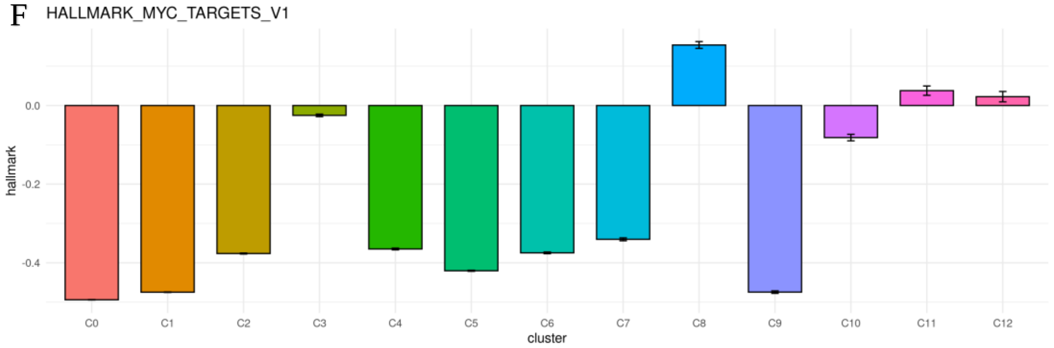
HALLMARK_MYC_TARGETS_V1
hallmark
0.0
-0.2
-0.4
C0 C1 C2 C3 C4 C5 C6 C7 C8 C9 C10 C11 C12
cluster

G
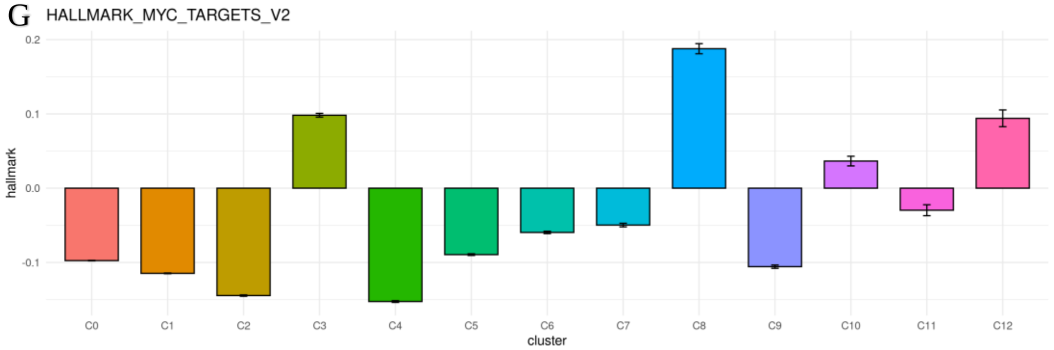
HALLMARK_MYC_TARGETS_V2
hallmark
0.2
0.1
0.0
-0.1
C0 C1 C2 C3 C4 C5 C6 C7 C8 C9 C10 C11 C12
cluster

H
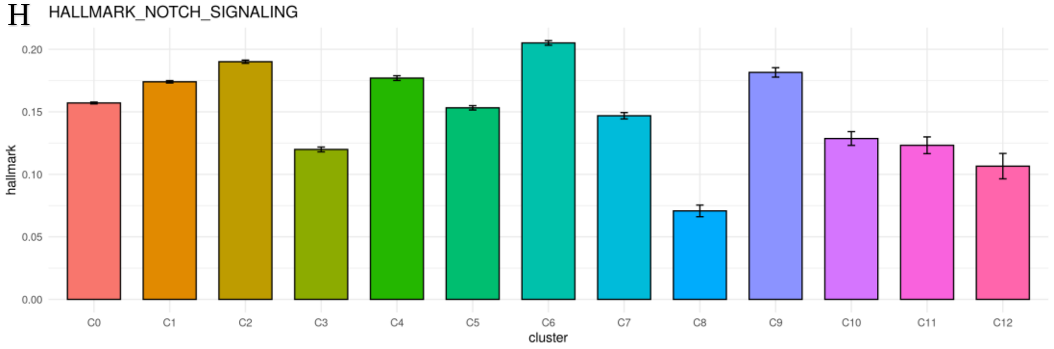
HALLMARK_NOTCH_SIGNALING
hallmark
0.20
0.15
0.10
0.05
0.00
C0 C1 C2 C3 C4 C5 C6 C7 C8 C9 C10 C11 C12
cluster

I HALLMARK_IL6_JAK_STAT3_SIGNALING

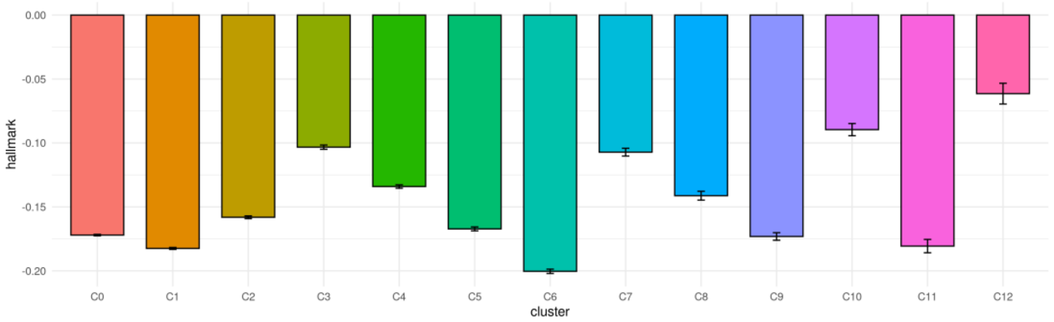

J HALLMARK_ALLOGRAFT_REJECTION

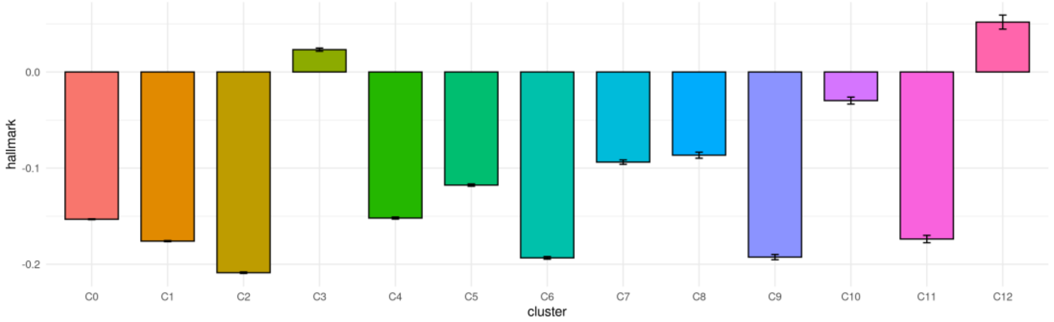

K HALLMARK_ANGIOGENESIS

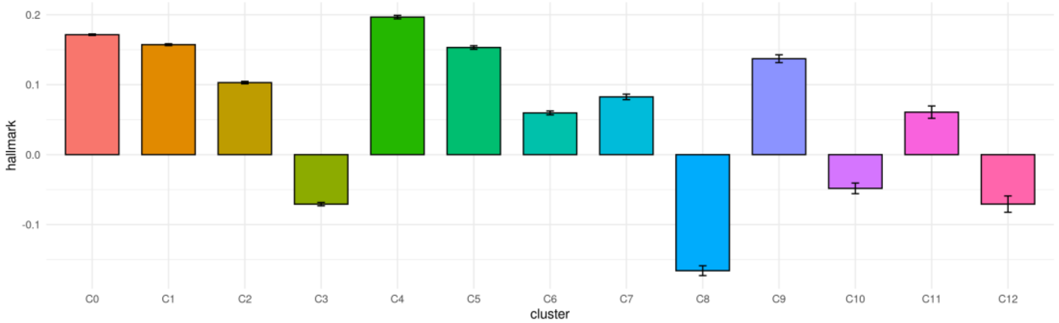

L

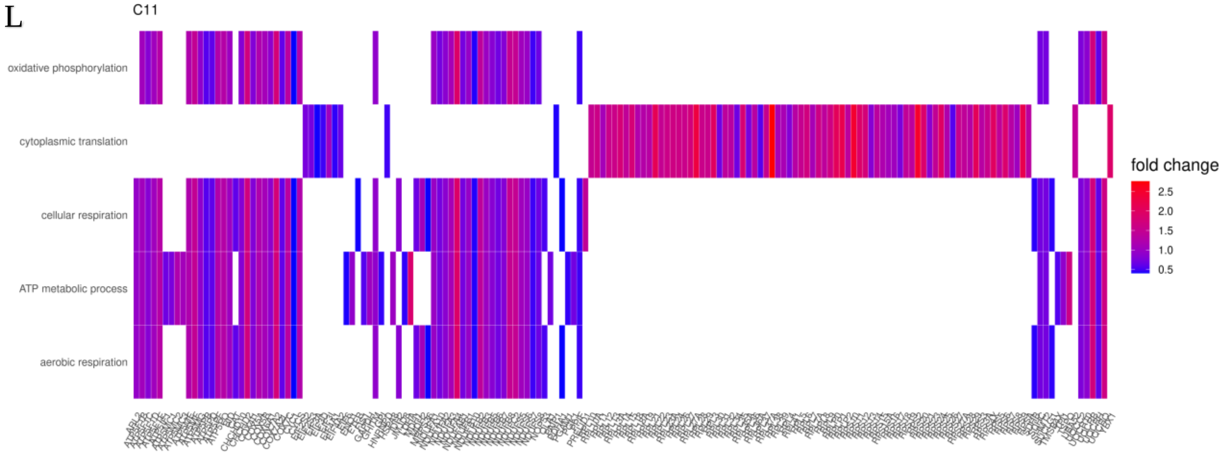

C11
oxidative phosphorylation
cytoplasmic translation
cellular respiration
ATP metabolic process
aerobic respiration
fold change
2.5
2.0
1.5
1.0
0.5

M

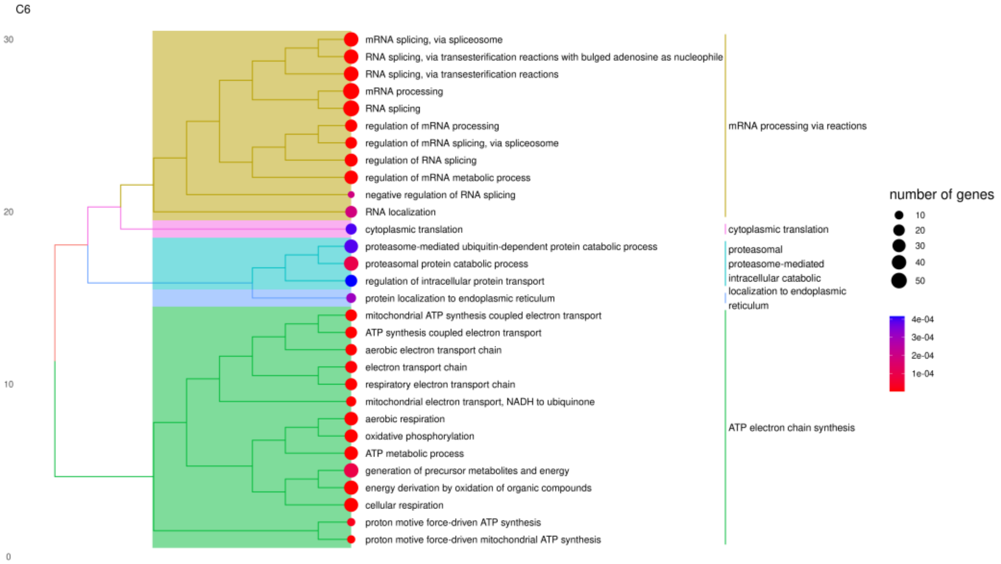

C6
30
20
10
0
mRNA splicing, via spliceosome
RNA splicing, via transesterification reactions with bulged adenosine as nucleophile
RNA splicing, via transesterification reactions
mRNA processing
RNA splicing
regulation of mRNA processing
regulation of mRNA splicing, via spliceosome
regulation of RNA splicing
regulation of mRNA metabolic process
negative regulation of RNA splicing
RNA localization
cytoplasmic translation
proteasome-mediated ubiquitin-dependent protein catabolic process
proteasomal protein catabolic process
regulation of intracellular protein transport
protein localization to endoplasmic reticulum
mitochondrial ATP synthesis coupled electron transport
ATP synthesis coupled electron transport
aerobic electron transport chain
electron transport chain
respiratory electron transport chain
mitochondrial electron transport, NADH to ubiquinone
aerobic respiration
oxidative phosphorylation
ATP metabolic process
generation of precursor metabolites and energy
energy derivation by oxidation of organic compounds
cellular respiration
proton motive force-driven ATP synthesis
proton motive force-driven mitochondrial ATP synthesis
mRNA processing via reactions
cytoplasmic translation
proteasomal
proteasome-mediated
intracellular catabolic
localization to endoplasmic
reticulum
ATP electron chain synthesis
number of genes
10
20
30
40
50
4e-04
3e-04
2e-04
1e-04

N

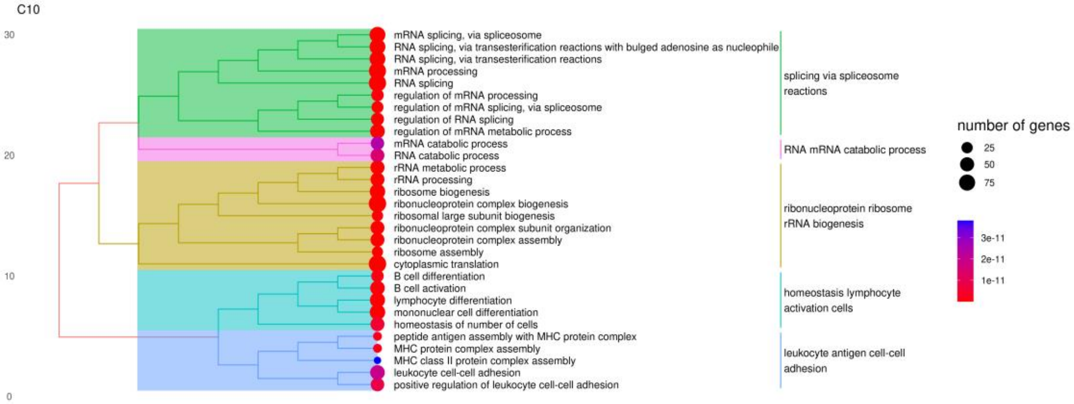

C10
30
20
10
0
mRNA splicing, via spliceosome
RNA splicing, via transesterification reactions with bulged adenosine as nucleophile
RNA splicing, via transesterification reactions
mRNA processing
RNA splicing
regulation of mRNA processing
regulation of mRNA splicing, via spliceosome
regulation of RNA splicing
regulation of mRNA metabolic process
mRNA catabolic process
RNA catabolic process
rRNA metabolic process
rRNA processing
ribosome biogenesis
ribonucleoprotein complex biogenesis
ribosomal large subunit biogenesis
ribonucleoprotein complex subunit organization
ribonucleoprotein complex assembly
ribosome assembly
cytoplasmic translation
B cell differentiation
B cell activation
lymphocyte differentiation
mononuclear cell differentiation
homeostasis of number of cells
peptide antigen assembly with MHC protein complex
MHC protein complex assembly
MHC class II protein complex assembly
leukocyte cell-cell adhesion
positive regulation of leukocyte cell-cell adhesion
splicing via spliceosome
reactions
RNA mRNA catabolic process
ribonucleoprotein ribosome
rRNA biogenesis
homeostasis lymphocyte
activation cells
leukocyte antigen cell-cell
adhesion
number of genes
25
50
75
3e-11
2e-11
1e-11

Supplementary Figure 2. Clustered platelets and their unique pathway expression changes. (A - K) Bar plot of hallmark gene sets expression among clusters. A) MYC_targets_v1, B)MYC_targets_v1, C) Oxidative phosphorylation D) Glycolysis E) Inflammatory response F) Interferon alpha response, G) Interferon gamma response, H) Notch signaling I) IL6/JAK/STAT3 signaling. (L) Heatmap display fatal cluster C11 up-regulated genes and enriched pathways in gene ontology (GO). (M, N) Tree plot display survival cluster C6 and C10 enriched pathways in GO.

A

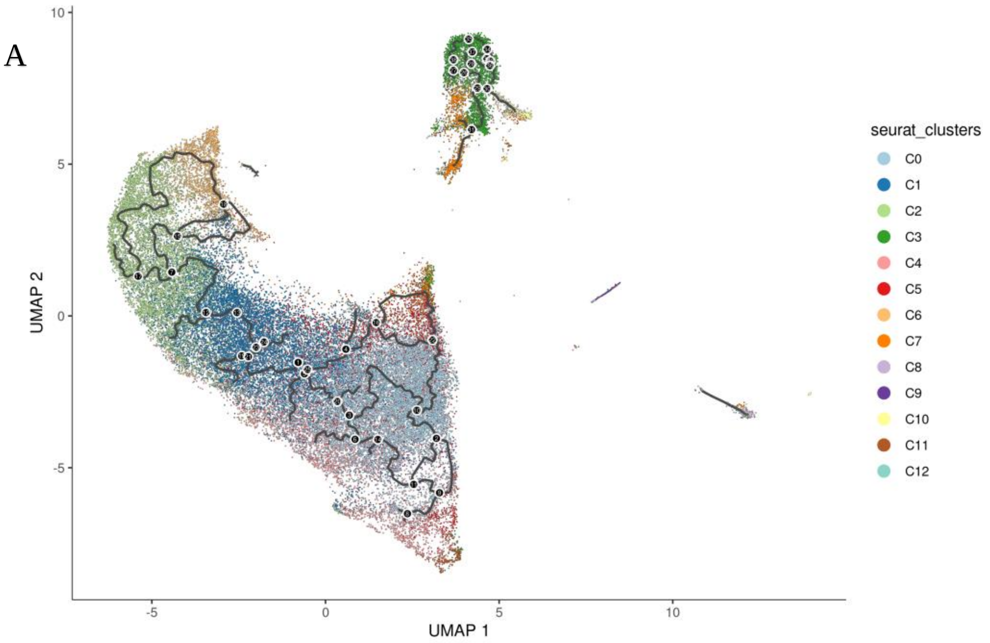

B

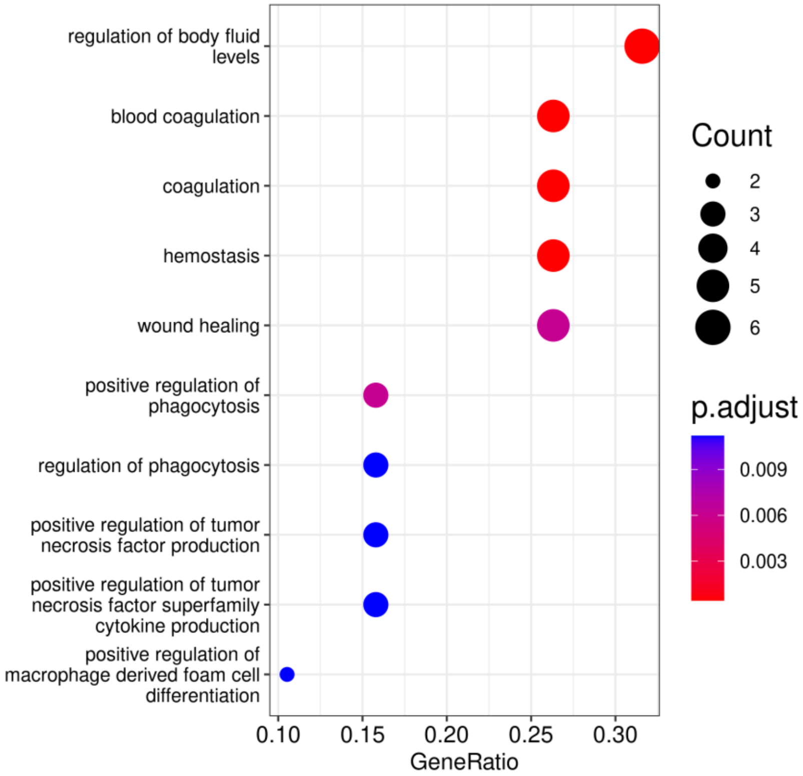

Supplementary Figure 3. (A) Pseudo-time plot of platelets from all clusters exhibiting trajectory fates. (B) The fatal gene module list enriched pathways from GO, KEGG, and Reactome databases. The circle size represents the gene counts included in the pathway; color represents the adjusted P value.

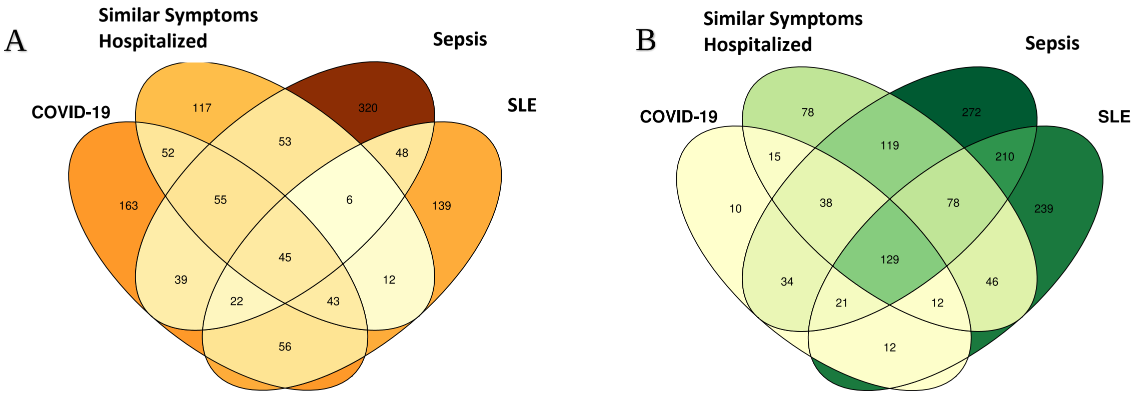

C

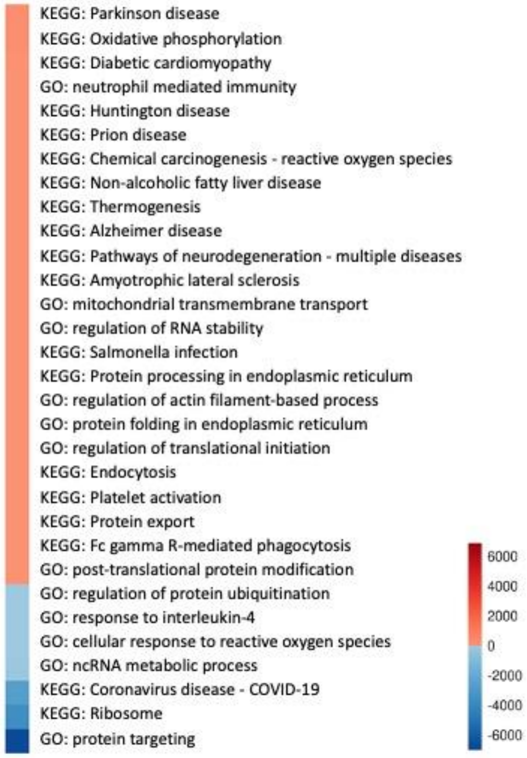

Supplementary Figure 4. **Platelets expression changes among healthy controls, sepsis, similar symptom hospitalized, COVID-19 and SLE patients**. Venn diagrams describing changes in differential expression genes (DEGs) from COVID-19, similar symptoms hospitalized (SSH), sepsis, and systemic sclerosis lupus (SLE) vs. healthy controls (HC), A) Up-regulated genes. B) Down-regulated genes. DEGs with adjusted p value below 0.05 and log2 fold change over 0.25 were used for generation of the VENN figure. (C) Heatmap illustration of COVID-19 and sepsis patients vs healthy contol pathways. Color are decided by the product of the COVID-19 and sepsis up/down-regulated enriched pathway log10 (adjusted p value). The GO terms were reduced to representative ones using Revigo [PMID: 21789182] (the cutoffs were similarity > 0.4) then overlapped the terms.

A

B

C

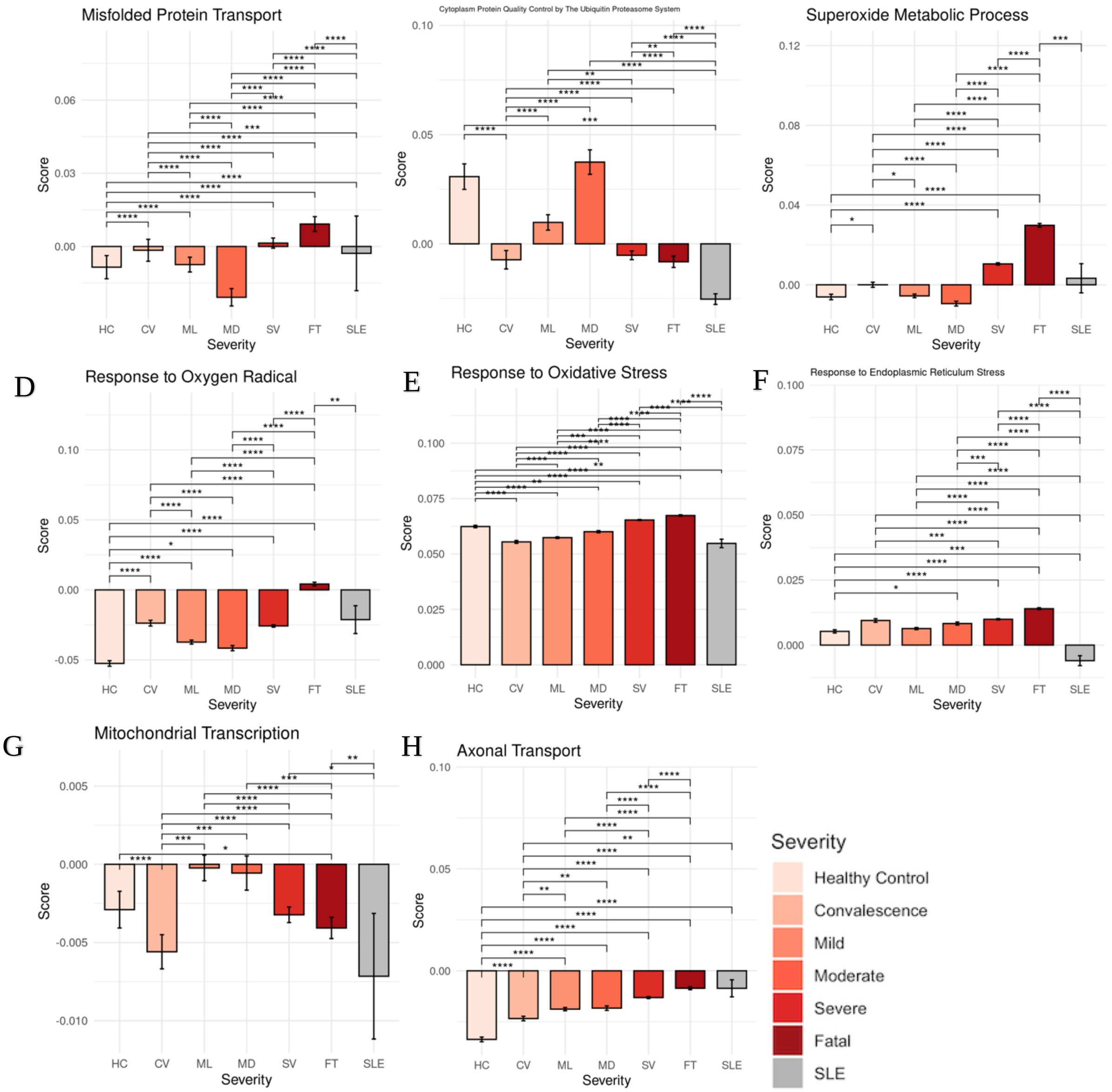

Supplementary Figure 5. **Platelet transcriptional changes under different disease severity related to neurodegeneration diseases**.

(A– H) Comparisons of pathway module scores across disease severities in platelets. The included modules contain genes related to **A)** Misfolded Protein Transport, **(B)** Cytoplasm Protein Quality Control by The Ubiquitin Proteasome System, **(C)** Superoxide Metabolic Process, **(D)** Response to Oxygen Radical, **(E)** Response to Oxidative Stress, **(F)** Response to Endoplasmic Reticulum Stress, **(G)** Mitochondrial Transcription**, (H)** Axonal Transport.

The differences in scores associated with adjusted *P*-values below 0.05, 0.01, 0.001, and 0.0001 are indicated as *, **, ***, and ****, respectively and not significant ones are not shown. The significance analysis was performed using Wilcoxon tests.

Clusters	Severity	Number	Frequency
C0	CV	1107	0.082
C0	FT	2876	0.214
C0	HC	743	0.055
C0	MD	1027	0.076
C0	ML	1959	0.146
C0	SLE	53	0.004
C0	SV	5688	0.423
C1	CV	879	0.083
C1	FT	2101	0.2
C1	HC	608	0.058
C1	MD	949	0.09
C1	ML	1839	0.175
C1	SLE	51	0.005
C1	SV	4102	0.39
C2	CV	561	0.073
C2	FT	1377	0.179
C2	HC	305	0.04
C2	MD	722	0.094
C2	ML	1338	0.174
C2	SLE	17	0.002
C2	SV	3373	0.438
C3	CV	81	0.021
C3	FT	202	0.052
C3	HC	636	0.165
C3	MD	712	0.184
C3	ML	962	0.249
C3	SLE	4	0.001
C3	SV	1267	0.328
C4	CV	137	0.043
C4	FT	1224	0.385
C4	HC	139	0.044
C4	MD	127	0.04
C4	ML	264	0.083
C4	SLE	4	0.001
C4	SV	1284	0.404
C5	CV	155	0.055
C5	FT	520	0.185
C5	HC	472	0.167

C5	MD	310	0.11
C5	ML	263	0.093
C5	SLE	10	0.004
C5	SV	1088	0.386
C6	CV	168	0.072
C6	FT	262	0.112
C6	HC	18	0.008
C6	MD	293	0.125
C6	ML	375	0.16
C6	SLE	11	0.005
C6	SV	1211	0.518
C7	CV	125	0.081
C7	FT	211	0.137
C7	HC	65	0.042
C7	MD	150	0.097
C7	ML	212	0.138
C7	SLE	13	0.008
C7	SV	764	0.496
C8	CV	370	0.36
C8	FT	86	0.084
C8	HC	57	0.055
C8	MD	15	0.015
C8	ML	38	0.037
C8	SLE	0	0
C8	SV	462	0.449
C9	CV	28	0.047
C9	FT	263	0.442
C9	HC	102	0.171
C9	MD	9	0.015
C9	ML	37	0.062
C9	SLE	0	0
C9	SV	156	0.262
C10	CV	51	0.105
C10	FT	62	0.127
C10	HC	36	0.074
C10	MD	8	0.016
C10	ML	42	0.086
C10	SLE	6	0.012
C10	SV	282	0.579

C11	CV	6	0.021
C11	FT	221	0.784
C11	HC	3	0.011
C11	MD	0	0
C11	ML	10	0.035
C11	SLE	0	0
C11	SV	42	0.149
C12	CV	27	0.158
C12	FT	9	0.053
C12	HC	21	0.123
C12	MD	8	0.047
C12	ML	20	0.117
C12	SLE	0	0
C12	SV	86	0.503

Supplementary Table 1. Number and frequency of each cluster of platelets composed of each disease severity.

Clusters	Outcomes	Number	Frequency
C0	HC	743	0.055
C0	FT	2876	0.214
C0	S	7135	0.53
C0	Unknown	2699	0.201
C1	HC	608	0.058
C1	FT	2101	0.2
C1	S	5527	0.525
C1	Unknown	2293	0.218
C2	HC	305	0.04
C2	FT	1377	0.179
C2	S	4325	0.562
C2	Unknown	1686	0.219
C3	HC	636	0.165
C3	FT	202	0.052
C3	S	2394	0.62
C3	Unknown	632	0.164
C4	HC	139	0.044
C4	FT	1224	0.385
C4	S	1106	0.348
C4	Unknown	710	0.223

C5	HC	472	0.167
C5	FT	520	0.185
C5	S	1190	0.422
C5	Unknown	636	0.226
C6	HC	18	0.008
C6	FT	262	0.112
C6	S	1675	0.716
C6	Unknown	383	0.164
C7	HC	65	0.042
C7	FT	211	0.137
C7	S	1020	0.662
C7	Unknown	244	0.158
C8	HC	57	0.055
C8	FT	86	0.084
C8	S	774	0.753
C8	Unknown	111	0.108
C9	HC	102	0.171
C9	FT	263	0.442
C9	S	156	0.262
C9	Unknown	74	0.124
C10	HC	36	0.074
C10	FT	62	0.127
C10	S	350	0.719
C10	Unknown	39	0.08
C11	HC	3	0.011
C11	FT	221	0.784
C11	S	45	0.16
C11	Unknown	13	0.046
C12	HC	21	0.123
C12	FT	9	0.053
C12	S	53	0.31
C12	Unknown	88	0.515

Supplementary Table 2. Number and frequency of each cluster of platelets composed of each outcome situation.

Cluster	Gene	avg_log2FC	pct.1	pct.2	p_val	p_val_adj

C0	MYL9	1.44979842	0.927	0.676	0	0
C0	FCER1G	1.3785031	0.695	0.431	0	0
C0	PARVB	1.36861383	0.659	0.433	0	0
C1	TMEM140	1.08200129	0.626	0.378	0	0
C1	PNMA1	1.05784379	0.356	0.22	0	0
C1	CDKN1A	1.04413495	0.608	0.41	0	0
C2	DEPP1	2.46460805	0.233	0.014	0	0
C2	INKA1	2.3932049	0.64	0.103	0	0
C2	FAM110A	2.21126078	0.805	0.225	0	0
C3	JUNB	3.28521179	0.807	0.137	0	0
C3	JUN	3.27880346	0.79	0.12	0	0
C3	RPL34	3.17377804	0.975	0.319	0	0
C4	HPSE	1.46464595	0.6	0.148	0	0
C4	WFDC1	1.41410805	0.354	0.058	0	0
C4	PF4	1.31987935	0.998	0.93	0	0
C5	JCHAIN	1.46558029	0.258	0.105	7.35E-164	9.60E-160
C5	TMSB10	0.31795166	0.651	0.491	7.48E-66	9.77E-62
C5	PSME2	0.28101928	0.076	0.104	1.21E-64	1.58E-60
C6	CRBN	2.57584885	0.717	0.227	0	0
C6	CD58	2.54909621	0.334	0.049	0	0
C6	SRSF3	2.50170105	0.639	0.197	0	0
C7	S100A8	2.77269395	0.73	0.434	0	0
C7	MALAT1	2.75406188	0.965	0.655	0	0
C7	NEAT1	2.34567652	0.645	0.245	0	0
C8	RPLP0	3.02681342	0.967	0.261	0	0
C8	RPS6	2.84360105	0.974	0.266	0	0
C8	RPS23	2.84118611	0.994	0.323	0	0
C9	HBB	6.9482949	0.576	0.212	0	0
C9	HBA2	6.53307512	0.45	0.084	0	0
C9	HBA1	5.67655265	0.501	0.101	0	0
C10	CD74	3.89201311	0.986	0.299	0	0
C10	HLA-DRA	3.41506579	0.899	0.094	0	0
C10	CD79A	2.77837866	0.674	0.019	0	0
C11	TPT1	2.24007978	0.996	0.635	1.13E-210	1.48E-206
C11	POLR2L	2.34141381	0.904	0.143	7.60E-195	9.93E-191
C11	CSRP1	1.94266214	0.798	0.093	3.92E-171	5.12E-167
C12	MALAT1	2.74811184	1	0.663	1.09E-122	1.42E-118
C12	RPL3	2.48307029	0.971	0.29	1.54E-117	2.02E-113
C12	RPS16	2.21622308	0.977	0.26	7.54E-106	9.85E-102

Supplementary Table 3. Up-regulated genes in each platelet cluster compared to other clusters of platelets.

COVID-19, SSH, sepsis, SLE compare to HC	
Overlapped up-regulated genes	Overlapped down-regulated genes
AP2B1	ANXA1
ATP5F1E	BTG1
ATP5MPL	BTG2
BEX3	CABP5
BRK1	CD3E
C9orf16	CD48
CASP4	CD52
CD226	CD7
CYTOR	COMMD6
DNAJC15	CORO1A
DSTN	CRIP1
DYNLRB1	CXCR4
EIF1	DDX5
FCER1G	DUSP1
GLA	EEF1A1
GNG5	EEF1B2
GPX4	EEF1D
GTF2A2	EEF2
HSPB1	EIF2S3
IFI27L2	EIF3E
IFITM2	EIF3F
IFITM3	EIF3H
ISCA1	EIF3K
KIFAP3	EIF3L
LCN2	ETS1
MAPRE1	FAU
NAA38	FOS
NAP1L1	GIMAP7
NDUFS5	GRHL1
OST4	GTPBP2
RHEB	HNRNPA1
RHOC	HNRNPDL

S100A8	HSPA8
S100A9	ICAM3
SERF2	IDS
SMIM27	IER2
STMP1	IL32
TBC1D15	IL7R
TIMP1	JUN
TMEM219	JUNB
UBL5	KLF2
UQCR11	LCK
XK	LDHB
YWHAE	LEF1
ZFAND3	LEPROTL1
	LIMD2
	LINC00861
	LITAF
	LSP1
	LTB
	MAL
	MALAT1
	MRFAP1
	NACA
	NOSIP
	NPM1
	ODC1
	PABPC1
	PFDN5
	PTPRC
	RACK1
	RGCC
	RPL10
	RPL10A
	RPL11
	RPL12
	RPL13
	RPL14
	RPL17
	RPL18
	RPL18A

RPL19
RPL22
RPL23A
RPL24
RPL26
RPL28
RPL29
RPL3
RPL30
RPL32
RPL34
RPL35
RPL36
RPL37
RPL39
RPL4
RPL5
RPL6
RPL7
RPL7A
RPL8
RPLP0
RPLP2
RPS12
RPS13
RPS14
RPS15
RPS15A
RPS16
RPS18
RPS19
RPS2
RPS21
RPS23
RPS24
RPS25
RPS26
RPS27A
RPS28

RPS3
RPS3A
RPS4X
RPS5
RPS6
RPS7
RPS8
RPSA
SARAF
SLC25A6
SNRPD2
SSR2
TCF7
TNS1
TOMM20
TOMM7
TSPAN18
TUBA1C
ZFP36L2

Supplementary Table 4. Consistently up/down regulated genes in COVID-19, similar symptom hospitalized (SSH), sepsis, and SLE patients compared to healthy controls (HC).
